# Basic Geo-Spatial Data Literacy Education for Economic Applications

**DOI:** 10.1007/s42489-023-00135-9

**Published:** 2023-04-06

**Authors:** Carsten Juergens, Andreas P. Redecker

**Affiliations:** Geomatics Group, Institute of Geography, Faculty of Geosciences, Ruhr University Bochum, Bochum, Northrhine-Westphalia Germany

**Keywords:** Spatial thinking, Map reliability, Data literacy, Trustworthiness, Geospatial teaching, Site allocation

## Abstract

Geospatial data literacy is of paramount importance in an increasingly digital business world. Especially in economic decision-making processes, the ability to judge the trustworthiness of pertinent data sets is inevitable for reliable decisions. Thus, geospatial competencies need to supplement the university’s teaching syllabus of economic degree programmes. Even if these programmes already have a lot of content, it is worth adding geospatial topics to educate students as skilled young experts, being geospatially literate. This contribution shows an approach on how to sensitise students and teachers with an economics background to understand the origin of geospatial data sets, their specific nature, their quality and how to gain geospatial data sets with a particular focus on sustainable economics applications. It proposes a teaching approach for educating students on geospatial characteristics of data, making them aware of spatial reasoning and spatial thinking. Especially it is vital to give them an impression of the manipulating nature of maps and geospatial visualisations. The aim is to show them the power of geospatial data and map products for research in their specific thematic field. The presented teaching concept originates from an interdisciplinary data literacy course geared to students other than geospatial sciences. It incorporates elements of a flipped classroom and a self-learning tutorial. This paper shows and discusses the results of the implementation of the course. Positive exam results imply that the teaching concept provides a suitable way to impart geospatial competencies to students belonging other than geo-related subjects.

## Abstract (de)

Geodatenkompetenz ist in einer zunehmend digitalen Wirtschaftswelt von zentraler Bedeutung. Gerade in wirtschaftlichen Entscheidungsprozessen ist die Fähigkeit, die Vertrauenswürdigkeit einschlägiger Datensätze zu beurteilen, unabdingbar für verlässliche Entscheidungen. Geodatenkompetenzen müssen daher die universitäre Lehre in wirtschaftswissenschaftlichen Studiengängen ergänzen. Auch wenn diese Studiengänge bereits über viele Inhalte verfügen, lohnt es sich, sie um raumbezogene Themen zu ergänzen, um die Studierenden zu kompetenten Fachleuten auszubilden, die über Kenntnisse zum Raumbezug verfügen. In diesem Beitrag wird ein Ansatz vorgestellt, wie Studierende und Lehrende mit wirtschaftswissenschaftlichem Hintergrund dafür sensibilisiert werden können, die Herkunft von Geodatensätzen, ihre spezifische Beschaffenheit, ihre Qualität und die Gewinnung von Geodatensätzen mit besonderem Fokus auf nachhaltige wirtschaftliche Anwendungen zu verstehen und zu hinterfragen. Es wird ein Lehr-Ansatz vorgeschlagen, der darauf abzielt, Studierende über die raumbezogenen Merkmale von Daten zu unterrichten und sie für räumliches Denken und räumliche Argumentation zu sensibilisieren. Insbesondere ist es wichtig, ihnen einen Eindruck von der möglicherweise manipulativen Natur von Karten und georäumlichen Visualisierungen zu vermitteln. Ziel ist es, ihnen die von Geodaten und Kartenprodukten für die Forschung in ihrem jeweiligen Fachgebiet aufzuzeigen. Das vorgestellte Lehrkonzept stammt aus einem interdisziplinären Data Literacy Kurs, der sich an Studierende anderer Fachrichtungen als der Geowissenschaften richtet. Es beinhaltet Elemente eines Flipped Classroom und eines Selbstlerntutorials. In diesem Beitrag werden die Ergebnisse der Umsetzung des Kurses dargestellt und diskutiert. Die positiven Prüfungsergebnisse deuten darauf hin, dass das Unterrichtskonzept geeignet ist, Studierenden anderer Fächer als der Geowissenschaften raumbezogene Kompetenzen zu vermitteln.

## Introduction

Today, to an increasing degree, sustainability is a key component in economic decision-making and analyses. The economy relies more and more upon environmentally friendly and sustainable production with a reduced ecological footprint. The one-sided perspective to base decisions solely on economic parameters is no longer adequate and responsible stewardship of available natural resources is inevitable. For sustainable decisions, environmental and social aspects need to be considered. Many economic and ecological factors (like real estate, resources, emissions, and people) are characterized by their explicit geolocation and their implicit spatial characteristic. According to the spatial composition of things, it is easy to realise the inherent importance of geospatial data in an economic context. This not only makes sense because of the surplus of information in spatially related data. Brain research (Curelli et al. 2022; Herweg et al. 2018; Brotons-Mas et al. 2006) implies that it is also beneficiary because of the way the human brain deals with information spatially. It helps us more easily discover causal relations within data and memorise issues in the learning process. Geospatial data are a specific kind of data that differs from conventional economic data. It facilitates location intelligence applications for economic purposes.

To reliably work with geospatial data, specific data literacy skills are essential. Here data literacy is ‘the ability to read, work with, analyse and argue with data’ (Bhargava and D’Ignazio 2015) and, advanced by Panetta 2016, ‘the ability to read, write and communicate data in context, including an understanding of data sources and constructs, analytical methods and techniques applied, and the ability to describe the use case, application and resulting value'. Geospatial data literacy extends this concept by also being skilled in dealing with the spatial characteristics of so-called geodata. This includes the creation and interpretation of spatial data visualisations in maps and similar representations.

As incorporating geospatial methods in economics education is a relatively new approach, it seems necessary to establish new interdisciplinary teaching concepts. The concepts follow the objective of educating economy students on geospatial characteristics to become specialists in the new field of spatial economy (e.g., Pászto et al. 2021; Pászto et al. 2020a, b). It enables them to gain the benefits of spatial thinking in research and learning (e.g., Zimmermanova et al. 2021; Jürgens et al. 2018). In this respect, spatial thinking means integrating environmental parameters in economic decision-making, providing a high potential for achieving sustainable, environmentally sound results (Zimmermanova et al. 2021). Once they know about the characteristics of geospatial data and their processing, the students will quickly discover the power of geospatial data and map-related visualisations in a sustainable economic context.

Potential applications among others in a sustainable spatial economy are:

- Site allocation–optimal location of production site saves/reduces a lot of commodities (e.g., Moos 2020),
- Logistics–optimizing the volume of cargo saving time and fuel (e.g., Guan 2008)
- Last-mile-logistics–optimal routing solutions to individual customers (e.g., Amazon or other logistics) (e.g., Boysen et al. 2021),
- Shared (or collaborative) economy businesses (e.g., Uber, Foodpanda, etc.)–depending on mapping services and location analytics. It is a business model that revolves around the peer-to-peer sharing of assets and services. The beneficiaries of the shared economy business model include service providers, product owners, end-users, and intermediate platform or marketplace providers connecting the two. (e.g., Barykin et al. 2021; Lahti and Selosma 2013),
- Reachability of shops and storage buildings (e.g., Scott and He 2012),
- Generation of innovative indicators from earth observation imagery to support economic forecasts (e.g., Juergens and Meyer-Heß 2021),
- Estimation of natural resources volume and its change with time (e.g., tree harvest (e.g., Sader et al. 2003) or storm damage (e.g., Menšík 2020) based on earth observation imagery analysis,
- Site-specific mapping and estimation of natural disaster damage and economic losses depicted from earth observation imagery (e.g., Voigt et al. 2007)

### The Nature of Geodata

As mentioned earlier, geospatial data have their specific particularities. Their most essential aspect is the link to the real-world space by coordinates. In addition to that, they can be linked to thematic content giving access to individual properties. Thus, a geo-object is defined by its coordinates and its specific individual properties. The latter can be qualitative or quantitative regarding the nature of the attributes. One can distinguish between points, lines and polygons related to the geometry of geospatial objects. Geospatial data are mainly represented by two different data models: vector data and raster data, depending on the data acquisition and/or processing. While raster data represent real-world objects by a matrix of raster cells of a given cell size, vector data represent the same objects by points, lines or polygons (Pászto et al. 2020a, b). For many map products, vector data are used because of their easier handling and smaller file sizes (Pászto et al. 2020a, b; Juergens 2020a, b).

Geospatial data originate from widely different sources. Most geodata sets are acquired by surveyors in the field or by image acquisition from flying platforms. Those rather traditional data are available from official geo-portals mostly.

Due to the ubiquitous availability of mobile devices, many kinds of user-specific data are also available. Such user-generated data stem from the field of volunteered geographic information (e.g., Rienow et al. 2022; Sui et al. 2012), where people transmit (mostly actively) specific data (e.g., on temperatures, rainfall, routing etc.). Those data sets are well-suited to add additional measurements in between a sparse official measuring network (e.g., weather stations). Sometimes such user-generated data are used without the knowledge of the originators. For instance, mobile devices transmit positions and individual information like social media communication or fitness tracker data. It is rather easy for others to capture those data and analyse them without the acceptance of the owners (e.g., Pászto et al. 2014). For such a form of data collection, the term unvolunteered geographic information seems to be much more appropriate (Juergens 2020a, b).

Collecting (un-)volunteered geographic information is a form of crowdsourcing based on mass data (big data). This data is of varying accuracy and correctness regarding the semantic content and the precision of localisation. It depends on the device used. The high timeliness of such data is beneficial over data originating from authoritative sources that acquire data at a far lower frequency. Many citizen science projects rely on (un-)volunteered geographic data to gain up-to-date knowledge from short-term acquisition intervals. The digital nature of today´s geospatial data supports such analyses and offers rapid dissemination and global access to vast data resources. In addition to that, numerous online services provide geospatially referenced data.

Depending on the geospatial data origin the semantic reliability can differ. There might be a difference between official geostatistical data and similar data provided by companies, especially if a certain intention could be assumed.

Geospatial data are very specific in their nature. Besides their different appearance (e.g., vector vs. raster) they could also be distinguished by their scale. Since the representation of digital data can be enlarged seamlessly on digital devices the original scale of the source data should be known, to be able to judge the level of detail of the source. For most geoprocessing steps, the applied data sets should be of a similar original scale to guarantee a reliable result. Coarse small-scale geodata sets in between large-scale geodata sets can dilute the resulting information. For instance, it is different if an analysis is calculated on data sets representing countries or smaller administrative units. The smaller the administrative unit the more regional details are present and vice versa the lesser on the country level. In the context of maps, this is related to necessary generalisations for visualisations on a smaller scale. Analysing large-scale national data sets together with data sets based on counties might result in misleading results if those results are presented at the county level instead of the country level, due to different levels of detail (Juergens 2020a, b).

Regarding geospatial data quality, it is of interest how the data sets are generated or captured. A measurement delivers a specific accuracy depending on the technical device used and environmental conditions during acquisition. Such devices are GPS receivers or thermometers, for instance. Besides considerations related to the accuracy of individual measurements, it is also of interest, if the location is representative or if any disturbances are possibly influencing the mensuration. Automatically transmitted data can also be affected by errors or missing data.

It is also of interest to consider the age or timeliness of the data sets. Information on this is found in the metadata about each data set. Geometries and semantic content might have changed towards younger data sets of the same type. It is also necessary to consider that some data sets are reproduced only over long time intervals while others are reproduced more frequently. This circumstance can also affect the analysis of geodata. However, depending on the research question it could be helpful to use older data sets to analyse a former situation and possibly compare it with a more recent situation. In the case of earth observation data, one could compare the real-world situation between different image acquisition dates and perform a change detection analysis to identify changes in the natural or man-made environment.

Areal representations are highly dependent on their map projection. Map projections follow different rules to reproduce the real three-dimensional world on a two-dimensional map. As a consequence of different map projections applied, identical land areas appear in different shapes. This becomes obvious in global, small-scale maps.

### Geodata to the Mind

GIS and web mapping technologies play an essential role in providing rapid visualisations of geospatial aspects within economic applications. But economists usually are not trained to read thematic maps critically. Cassettari (2021) compares maps with food. Buying food in a supermarket could be risky, and people, therefore, consider the ‘best before’ or ‘use by’ dates. Geospatial data should offer their sources as well as quality. Mostly, this is not the case. Cassettari (2021) points out that the quality of geographic data (concerning accuracy, completeness, consistency and currency) like all other data is inherently uncertain. The clear and neat appearance of thematic maps and other geo-visualisations does not give any reason for suspiciousness. Thus, with this appearance, misleading map-making could result in misinformation or misguidance of the map user or decision-maker. Wrong or weak decisions because of such information could be very costly in an economic context. Due to research on the usability and readability of thematic maps, rules are developed for their suitable and most importantly true appearance. For instance, Bertin (1967), Dibiase et al. (1992), MacEachran (1995) and Tyrner (2010) defined and complemented rules for proper choropleth maps. These rules determine how objects from our complex world should be transformed into maps that offer a simplified and standardised representation of reality. Map makers use symbols and signatures to describe complex objects from reality in a classified way to produce easy-to-read maps. A complimentary legend explains the map content with its specific symbology.

The cartographer Monmonier (1996) published a well-known book under the provoking title ‘How to lie with maps’. It intends to make people aware of the power of maps in general and the influence of cartographers while creating a map. Based on variations in scale, colours, line or text styles as well as symbol styles the appearance of a map can be influenced. An early list of seven visual map variables was developed by Bertin (1967): position, size, shape, orientation, colour, value, and texture. This was then extended by Morrison (1974) to nine visual variables, and later MacEachran (1995) expanded this list to twelve visual map variables: [(1) location, (2) size, (3) shape, (4) orientation, (5) colour hue, (6) colour value, (7) texture, (8) colour saturation, (9) arrangement, (10) crispness, (11) resolution, and (12) transparency] (Roth 2017). Map design is related to the communication of results stemming from spatial analysis. Thus, the readability and consequently the intuitive interpretation or misinterpretation of a map could be affected by the appearance of map variables used for the specific map design.

Mooney (2020) and Griffin (2020) illustrate the power of thematic maps. They show how to create trustworthy thematic maps avoiding misleading representations of geospatial data and possible misinterpretations. Regarding geospatial data literacy aspects in detail, Juergens (2020a, b) describes and illustrates many influencing factors. Some of them are:

- Level of generalization: the complexity of the real world needs simplification for its presentation in a classified depiction of a map. Thus, the cartographer influences the resulting map content by deciding what objects should be emphasised or repressed or which should be excluded from or included in the final map.
- Map projection: depending on the selected map projection, distortions of distance, direction and/or area are inevitable and need optimisation concerning the map’s purpose.
- Scale and level of detail: here, also a relation to generalization is obvious. For instance, map generalization is necessary to reduce the semantic details of maps to ensure their readability.
- Classification method with choropleth maps: the choice of a classification method and the number of thematic classes influence the information that can be extracted from a map.

It seems to be obvious, that maps used for the presentation of study results can be misleading themselves. In addition to that, geospatial data capture and subsequent geospatial information extraction can as well suffer from shortcomings and hinder trustworthy geo-processing and geo-visualisation.

### Geodata Literacy for Economics Students

Due to the paramount importance of geospatial data literacy in sustainable economic decision-making processes, it seems inevitable to introduce a tailored teaching approach. After all, it makes sense to increase economy students’ awareness of spatial reasoning and to introduce them to the power of geospatial data and map products for their future business applications. Of course, it will be impossible to teach all aspects of geomatics within an introductory class. But it should be focused on the most relevant aspects. One example of an interdisciplinary teaching approach is the ERASMUS + project SPATIONOMY (“Spatial exploration of economic data—methods of interdisciplinary analytics”) (e.g., Pászto et al. 2021; Zimmermanova et al. 2021; Jürgens et al. 2018). In this project, an interdisciplinary team of researchers and academic lecturers developed and tested a new teaching approach based on the concept of playful learning. The aim was to convey interdisciplinary methodological skills to students at the interface between geomatics and economics. Besides teaching basic knowledge in the two subjects, central elements of the teaching concept are case studies and a simulation game with interdisciplinary challenges. At this, students apply the basic knowledge they gained during the preparatory phase to solve several rounds of game-based learning (Zimmermanova et al. 2021).

The SPATIONOMY approach delivers a valuable complement to the study programme of economics students. For them, it provides the opportunity for a deeper insight into the handling and the potential of geospatial data. But already, this format is too extensive and timely and can only provide an additional offer. There must be a far less time-consuming way to educate economic students effectively on the most relevant aspects of geospatial data literacy. The objectives for such a basic introduction must differ from the much more specialised and profound geomatics or geoinformatics study programs. Otherwise, the economics students would have to study an additional subject—which is not the aim of this proposition. Therefore, it is crucial to restrict the geospatial content to the very but substantial essentials.

Such learning objectives related to geospatial data literacy capability for economics students to support reliable decisions are:

- Knowledge of geospatial data characteristics and limitations
- Overview of (cost-free) reliable and open geodata sources
- Essential geo-processing knowledge, e.g., site allocation analysis
- Optimal spatial decision-making with economic and sustainability constraints
- Map-making principles
- Proper geo-visualisation of results for specific target groups
- Critical awareness of potential geodata manipulations as well as the power of map products
- Practical experience in geospatial data analysis.

## Materials and Methods

The following will outline the concept of an introductory teaching unit about geospatial data literacy for students not studying a geospatially related subject. It is part of an interdisciplinary course about data literacy in general held at the Ruhr-University Bochum, Germany. All units of this course cover different aspects of data literacy. They highlight the application of data in various disciplines like sociology, linguistics, law, natural sciences and many more. The preparations related to the course started before COVID-19 appeared. However, its appearance indicated a higher need for digital learning modules. During winter semester 2020/21 and the following summer semester of 2021, we used and evaluated the course concept outlined here.

The basic concept of the course was developed together by the involved scientists of the different subjects. It not only benefits from interdisciplinarity regarding the content but also from multiple different subject-specific experiences regarding teaching methods. Hence, the blueprint for this teaching unit is an interdisciplinary data literacy course established and designed by a group of experts from diverse scientific fields, each contributing its own scientific and didactic perspective. These were discussed and combined into the teaching concept incorporated here. Thus, the underlying concept is the outcome of an intense discussion within an interdisciplinary team of experts.

Every single unit is prepared and held by the expert of the respective scientific field within the framing of the whole course.

**Fig. 1:**
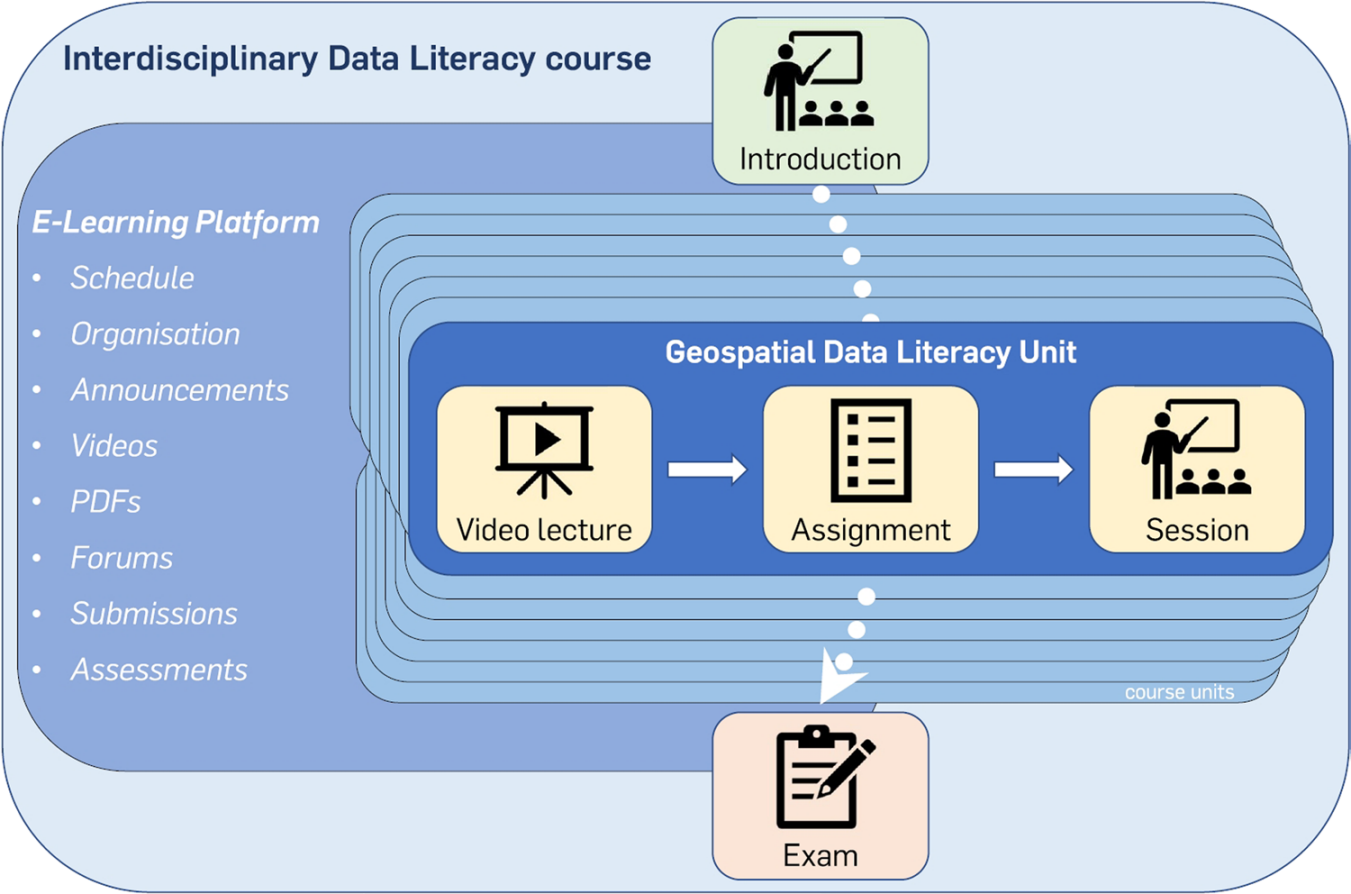
Structure of one thematic unit (e.g., the geospatial data literacy unit) within the whole interdisciplinary data literacy course (Source: Authors)

The course design follows the concept of blended learning with elements of a flipped classroom (DeLozier and Rhodes 2017; Milman 2012; Rotellar and Cain 2016) supported by an e-learning platform. Each unit of the course consists of three parts (compare Fig. 1):

- An asynchronous video lecture introduces the participants to the basics of the respective topic. Each lecturer prepares and records a continuous video, equivalent to the content and duration of a usual lesson. Students are supposed to watch it until the session that deals with that topic.
- An assignment task connected to the topic allows students to gain first experiences with the respective methodology and its methods and tools. The results are submitted and evaluated to form the rating for this unit.
- During the session, the students meet the respective lecturer personally. It takes place in a seminar room or via video conferencing. This allows for discussing and deepening the contents of the video lecture. Presented examples and projects give students an impression of how to apply the methodology shown—and what can be done (wrong) with it.

The provisioning of the units and their single parts follows a strict schedule. It is controlled by an e-learning platform that also receives the student’s submissions and supports keeping the deadlines.

Single forums assigned to each unit on the e-learning platform offer support and exchange among students and lecturers. It allows asking questions that arise while dealing with the task in particular. Answers can be given by lecturers and fellow students, respectively. In this way, given replies are available for all participants.

The three parts of the geospatial data literacy unit may serve as a blueprint for the proposed education of economic students on the most relevant aspects of geospatial data literacy. The presented methodology focuses on a teaching concept, not on statistical or geospatial methods themselves. It deals with the challenge of covering the essential basics of geomatics within a dense format. It must be capable of imparting enough knowledge to grasp the idea of spatial thinking for non-geospatial students.

### The Video Lecture

The video introduction to geospatial data literacy is divided into six successive sections. It was designed and recorded by one of the authors.

**Fig. 2:**
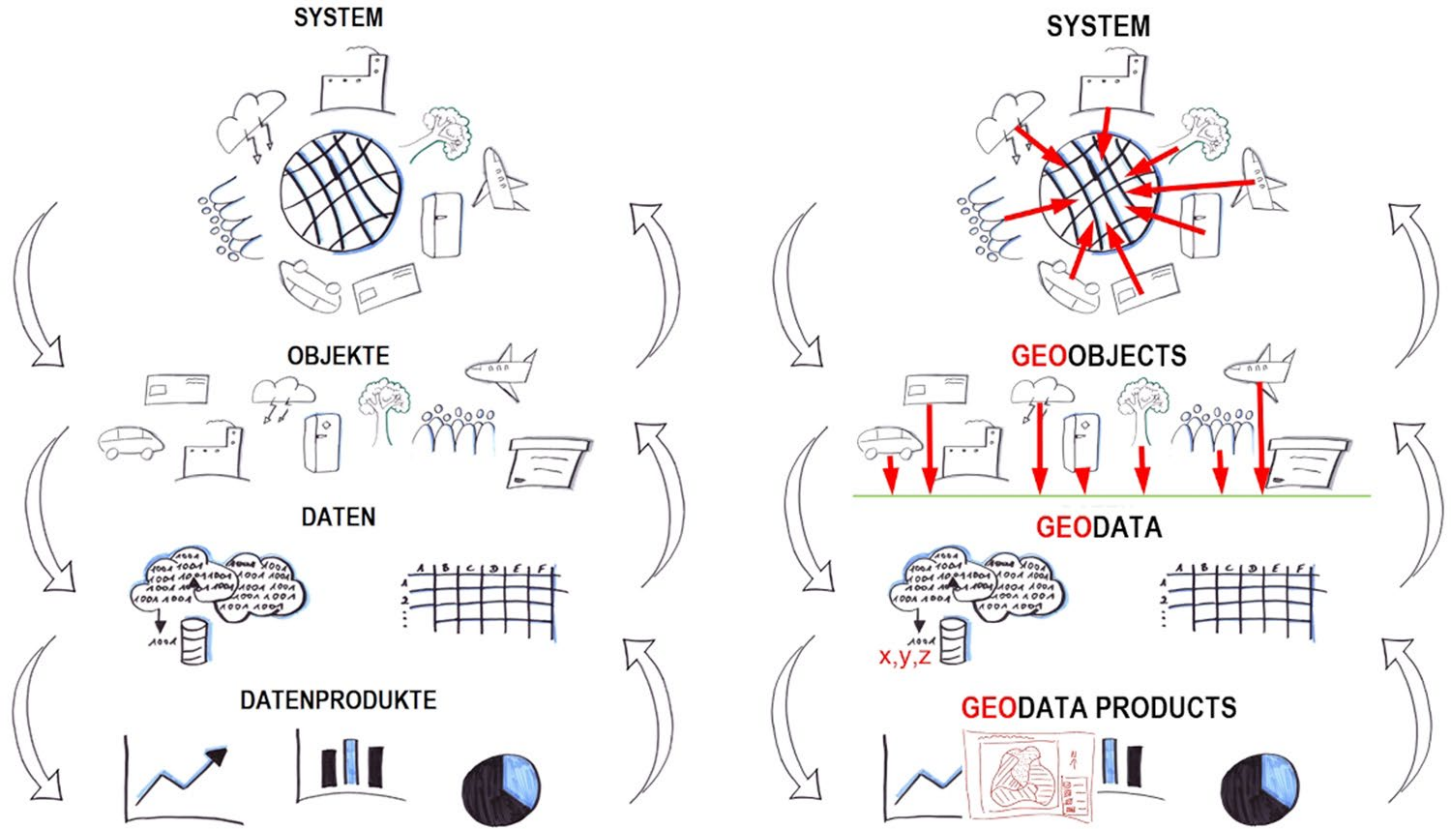
Adding the geo-aspect to the process model of added value from data (Source: Schüller et al. 2019, modified)

In the first section, the video lecture embeds the geo-aspect into the process model of added value from data (referring to Schüller et al. 2019). It forms the basis of the whole series of teaching units and provides a perfect frame to show the benefits of geospatial over non-geospatial data in a methodological context (Fig. 2).

A well-known citation from Fornefeld et al. (2008)—stating that about 80% of information has a spatial relation that can give added value in conjunction with the information itself—and an example about a spatial analysis of Twitter posts (Pászto et al. 2014) underlines the methodological contemplation.

After this, the key characteristics of geodata are explained:

- Geometry, defining the spatial relation of objects,
- Topology, the rules for the spatial relationship among objects,
- Topic, the required attributes of objects,
- Dynamics, the optional change of objects over time.

“From Earth to Plane” is the next section’s title that deals with projections, grids, datums and coordinates. It gives an impression of the basic problem and the necessity to transform spherical surfaces into flat ones that use metric coordinates instead of geographical or spherical ones.

Then the basic data models for geospatial information are introduced:

- Vector, representing objects as a collection of points, occasionally connected to lines or polygons
- TIN, a representation of surfaces by triangles formed from height points connected by lines
- Raster, representing an extract of the earth’s surface by values in a matrix of uniform squares
- Networks, composed of nodes and edges to represent networks for traffic, energy, fluids or other objects to move along.

**Fig. 3:**
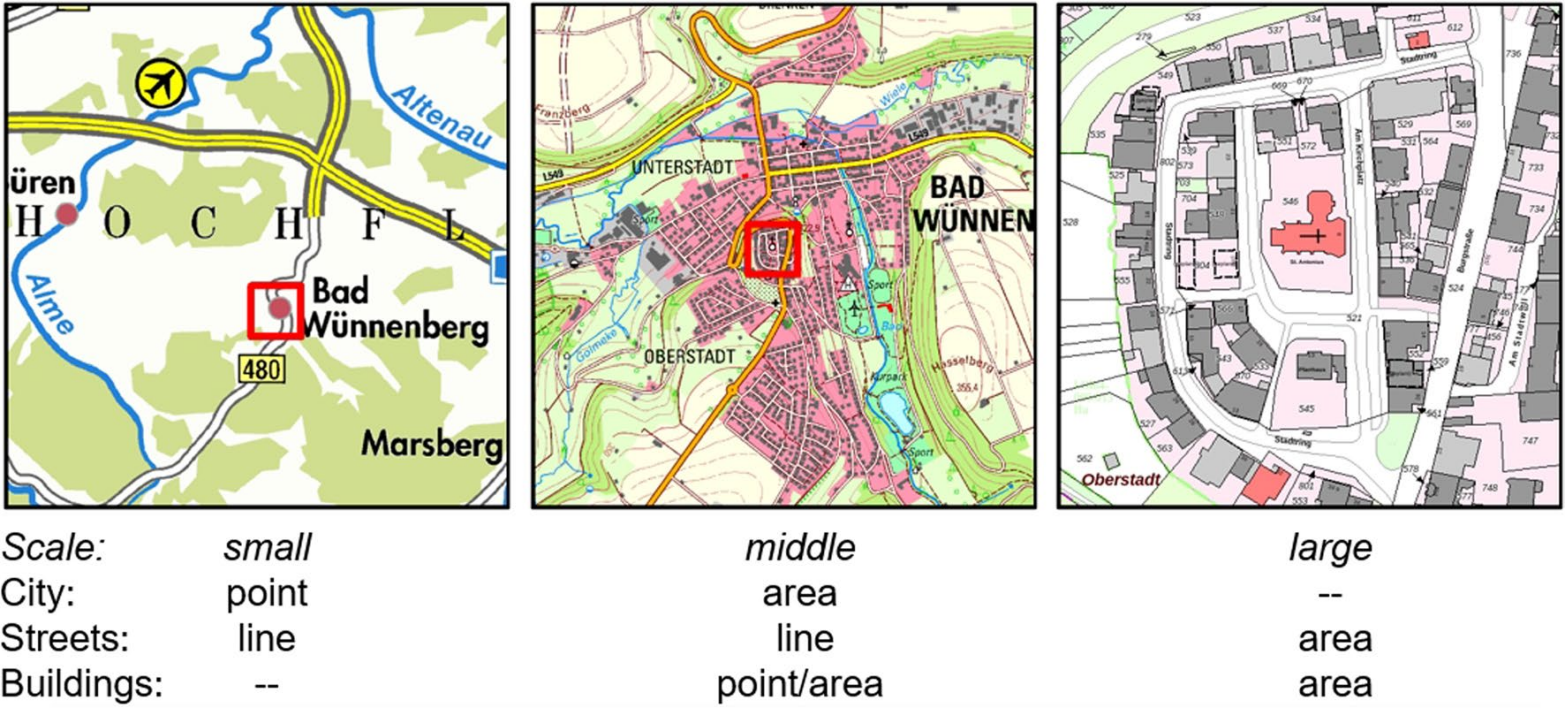
The geometric appearance of landscape features modelled at different dimensions depending on the scale (Data source: Geobasis NRW 2022a)

In this context, the scale dependency associated with the choice of geometric dimension within the vector model is also explained (Fig. 3).

Under the title “from the real world into the computer”, the following section deals with basic data formats for geospatial data. They allow storing data of objects in conjunction with their localisation in the real world.

The major types of these formats encompass.

- Files and databases for vector data,
- Simple files like csv-, txt- or xml-files for tabular data,
- Image file formats for raster data

Also, web services are introduced to provide and obtain geodata. A simple overview of software and services deals with applications that incorporate or provide geodata.

In this context, linking and joining tabular data to geospatial datasets is explained as an opportunity to localise data with implicit spatial reference. Regarding raster data formats, the importance of lossless compression for imagery data gets discussed by demonstrating the repercussions of lossy codecs.

**Fig. 4:**
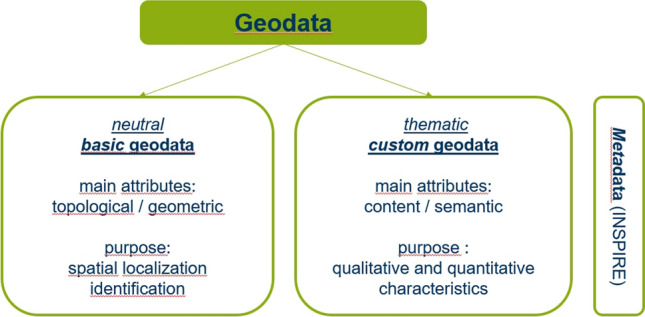
Types of data sources (Source: Authors)

The section “data sources” initially deals with the different origins of geodata and the primary discrimination of basic and custom geodata (Fig. 4).

After this, a few major suppliers of geodata are introduced: Authoritative and commercial vendors but also volunteered geographic information (VGI). This part is finalised by addressing the important aspect of data quality—especially with its specific implications regarding geodata.

**Fig. 5:**
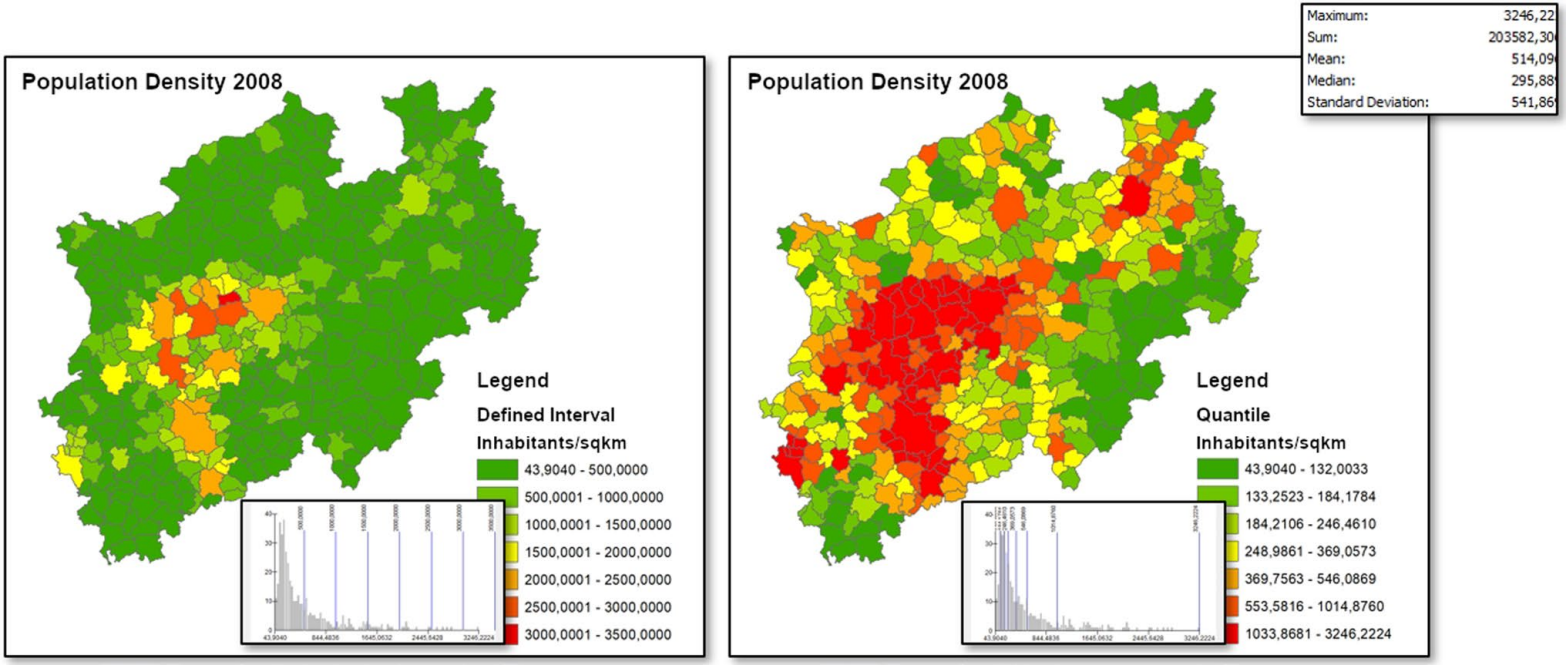
Impact of classification in choropleth maps (Source: Authors)

“Visualisation” is the title of the next section. It deals with the possible manipulating nature of maps. Examples of choropleth maps using different classification methods for the same data or choropleth maps from the news representing absolute numbers are used to present sources of miscommunication by maps (Fig. 5).

The video lecture closes with a “conclusion”, summarising the demand for.

- Spatial awareness,
- Spatial thinking and
- Spatial reasoning

as preconditions for literate working with geodata.

### The Task

**Fig. 6:**
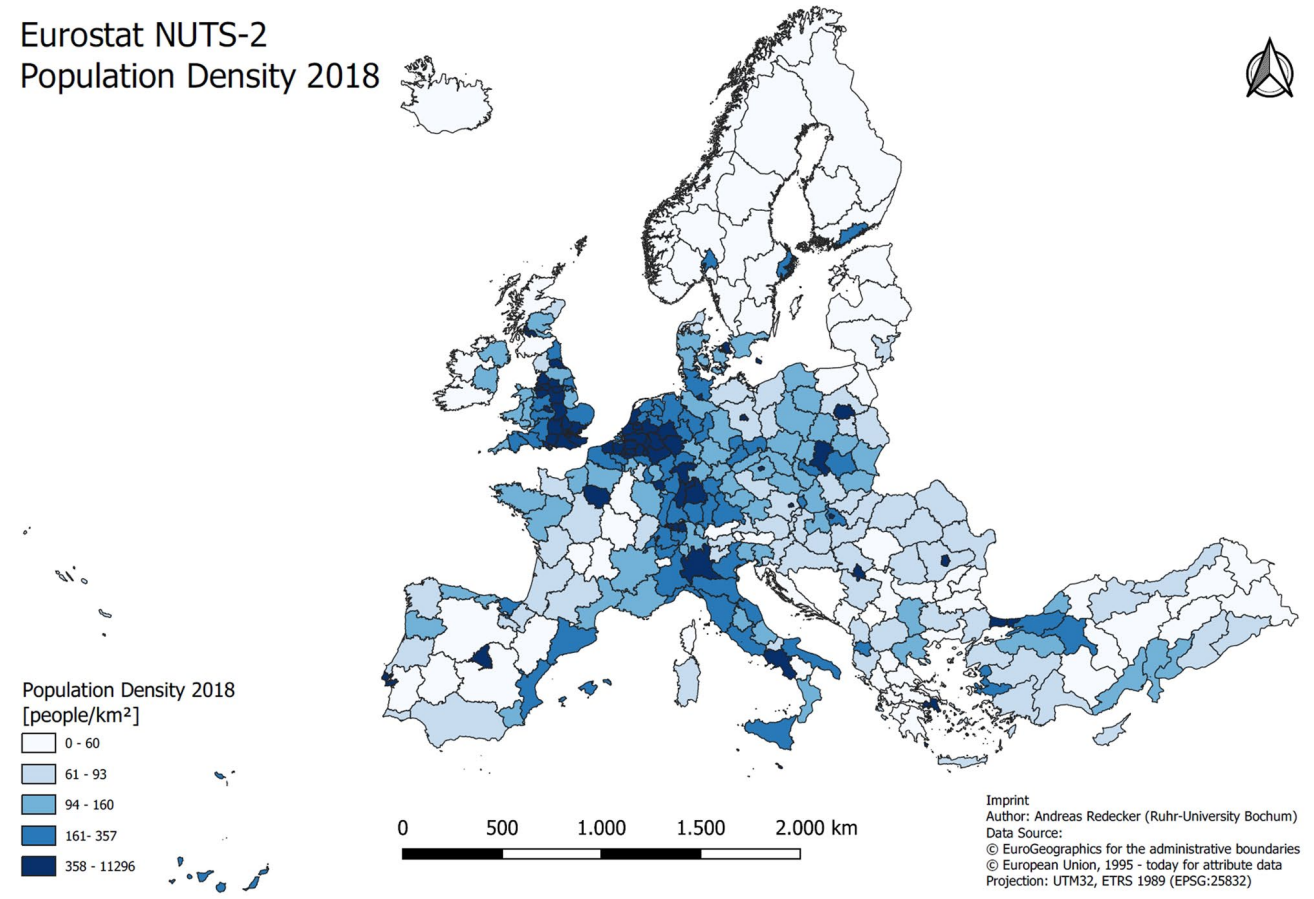
Example for the map of the population density in the NUTS-level 2 regions (Source: Authors; Data Sources: EuroGeographics: Eurostat/Gisco 2022, European Union: Eurostat 2022)

This part of the teaching unit allows the students to get their hands on geodata themselves by creating a map of the population density in NUTS-level 2 regions in Europe (Fig. 6). It consists of a video tutorial supported by a script-pdf and a finalising task. First, the tutorial leads the participants through the process of obtaining geodata and statistical data from the Eurostat web portal. They employ the open-source geo-information system (GIS) QGIS to process the required datasets and link the statistical information to the geodata. After calculating the population density, a choropleth map has to be created and finished in a map layout. Finally, the students submit their resulting PDF via the e-learning-system.

### The Session

In the session where students meet the lecturer—either in a seminar room or in a video call—they get the opportunity to discuss the contents of the video lecture. In addition to that, based on the fundamental knowledge provided beforehand, essential functions and operators of geospatial analysis get taught:

- Selection by Attribute and by location
- Buffering and Dissolve
- Clip, Difference, Symmetrical Difference, Intersect and Union
- Optimal Route, Travelling Salesman, Service Area, Location Allocation, OD-Matrices

**Fig. 7:**
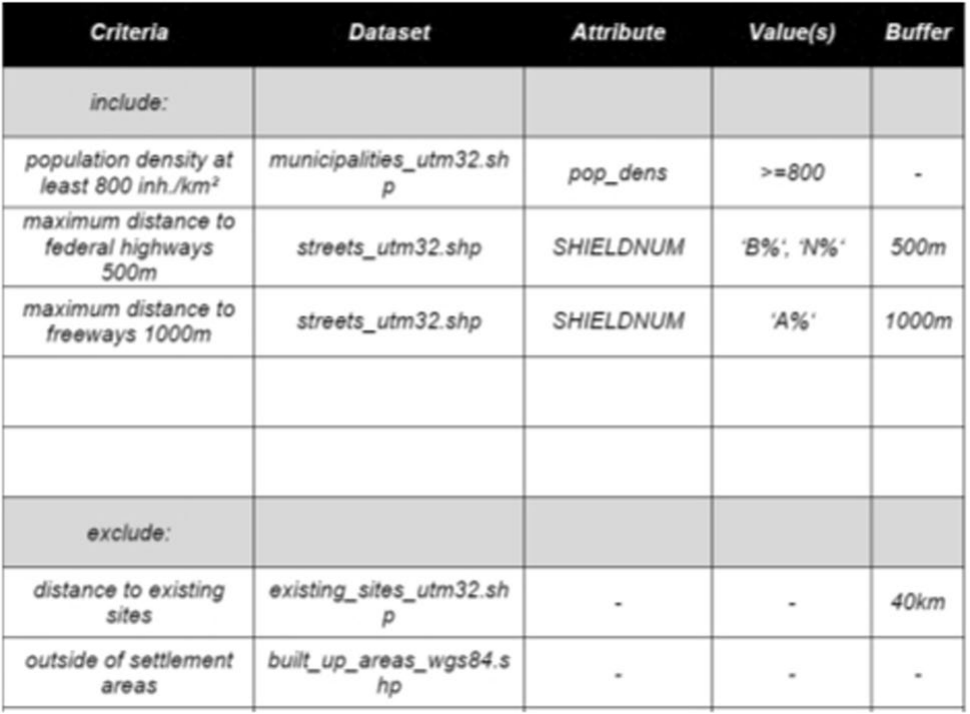
List with the required conditions related to individual datasets, attributes, desired values and optionally required buffers (Source: Authors)

Finally, an example explains the application of some tools. It shows a geospatial analysis for location allocation by systematically checking all conditions that need to apply to a suitable location on the one hand and those that must not apply on the other hand (Fig. 7). For this, the underlying task gets explained by talking through all conditions. Then they get related to the available data sets to identify the relevant properties and characteristics within the attribute data of the geo-objects.

**Fig. 8:**
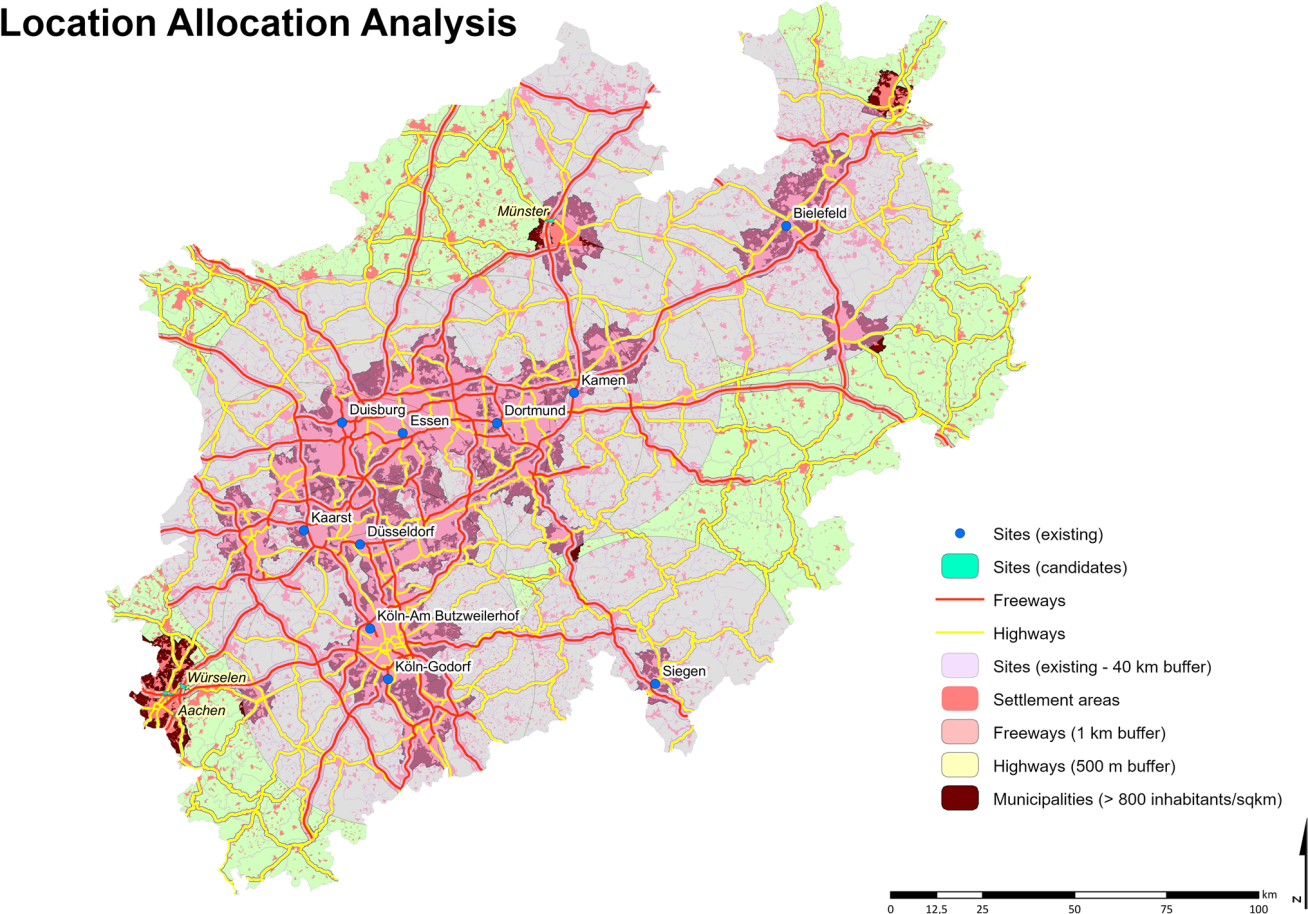
Map showing all input datasets and the result of the analysis (Source: Authors; Data Source: Geobasis NRW 2022b)

After applying all necessary operations using a geographic information system like selections, buffers, union, intersect and dissolve and looking at the results of each step the final result can be visualised in a decent map (Fig. 8).

## Results

About 60–80 students took part in the data literacy course in two semesters. Table 1 gives the numbers on how they performed in the overall course and the geospatial unit in particular.

**Table 1:** Participation and results of the data literacy exam and the geospatial unit in detail

Students	Semester 2020/21	2021
Participating in data literacy course ^1^	62 (100%)^2^	66 (100%)^2^
Visiting geospatial synchronous session	34 (54.8%)^2^	40 (60.6%)^2^
Submitting results	61 (98.4%)^2^	N/A
Participating in exam ^3^	58 + 3 (98.4%)^2^	60 + 4 (97.0%)^2^
Passed exam ^3^	57 + 3 (98.4%)^4^	59 + 4 (98,4%)^4^
Passed geospatial part of exam ^3^		
Question 1	48 + 1 (80.3%)^4^	45 + 15 (93.8%)^4^
Question 2	32 + 25 (93.4%)^4^	54 + 1 (85.9%)^4^

^1^Showing active participation on the e-learning platform

^2^Percentage based on the number of course participants

^3^First + second exam

^4^Percentage based on participants in the exam

In addition to the figures covering participation and exam results, various observations have been made that are beneficial for evaluating the geospatial data literacy unit described.

The first contact between the students and the lecturer came up via the forum on the e-learning platform. Compared to the number of participants, only a few of them asked for support in conjunction with the task.

Most of the questions arose from misunderstandings of single steps in the task or well-known careless mistakes while preparing data (taking care of data types or decimal separators, etc.).

In some cases, the questions unveiled a lack of basic skills in using standard features of operating systems or standard office software. Also, in every cycle, one or two students could not get the required open-source software running. Although it is available for the three prevalent operating systems (Windows, macOS and Linux). Few of them claimed to lack the appropriate equipment.

More general problems came up because of sudden changes in the functionality or structure of the Eurostat website. These changes made it difficult to find the required datasets or to configure the download in the way that is needed for further data processing within the task. This demanded a higher degree of transfer activity from the students, which not everybody was able to cope up with. These issues were solved by providing the necessary workarounds or updated instructions via the e-learning platform.

Not all students took the chance to meet personally at the session. About half of the participants turned up. Only a few of them joined in the discussion. Generally, they asked questions to confirm or extend their perception of the topic. None of them expressed doubts about the significance of the geospatial data literacy unit itself. Some of the participants requested information about opportunities to attend more extensive GIS-related courses.

## Discussion

The participation rate in the exam at 98.4% and 97.0% of all course participants is noticeably high. The success with a passed exam of 98.4% in both courses is also very good. The achievement ratio of the two geospatial questions is lower but still high (80.3% to 93.8%, depending on the year and the question).

The lack of basic skills with few students in using standard computer features is also well-known in other GIS introductory courses. It indicates a general demand for taking care of methodological computer literacy within or before study programmes. Therefore, it lies without the scope of a single course.

Regarding the lack of appropriate equipment, it is doubtful how the students concerned manage their courses anyhow. Since the University lends notebooks to students who can prove their needs, this should not be an issue. These doubts also apply to those with a severe lack of computer literacy.

The changes in a data-providing web portal are a known challenge for lecturers dealing with current data sources. Therefore, the instructions of tutorials including steps like this need inspection and most likely adaption before every iteration of the course. This causes some effort that may appear deterrent. One could also think about providing a “ready-to-use” data set. But the experience of searching, retrieving and integrating data from an external source would be lost. Dealing with data providers is a fundamental part of data analysis and didactically a valuable element in a course like this that should not be omitted.

The reduced attendance and the even lower participation in the synchronous session correspond to the usual experiences, especially with online lectures. Active participants are mostly well prepared, join in and enrich the discussion about the potential of geospatial aspects in data analyses. Moreover, they are interested in further learning perspectives regarding geospatial methods and skills. In accordance with high numbers of positive exam results, those who did not show up were able to cope with the asynchronous components of the unit independently—with no need for further assistance.

## Conclusion

This paper focuses on a teaching approach to educating non-geo-related students on fundamental principles of geospatial analyses. Furthermore, it underlines the power of geospatial data and map products in their specific thematic field of application.

Sustainable geospatially based economic decisions require training economics students in the fundamental principles of geospatial reasoning. With a high degree of geospatial data literacy, they can contribute to decisions considering the spatial components inherent in sustainable decision-making processes. Very often maps and map-like geo-visualizations are the results of a complex geo-processing procedure relying on many variables. Their outcome delivers the basis for eventually far-reaching decisions or investments. Thus, the result of geospatial analyses like geospatial data products and their visualisation in maps are of great importance.

The presented teaching concept has proved to be beneficial for training non-geo-related students on geospatial data literacy and spatial thinking. It imparts their awareness of the benefits, specific characteristics and the power of geospatial data. Economics students are future decision-makers at diverse positions in society and demand skills for considerate decision making especially in the context of sustainability. The outlined teaching concept provides a low-level entry to the topic of geospatial data literacy education. But it needs augmentation by elements supporting students with a lack of computer literacy on the one hand. On the other hand, courses for extending skills in geospatial data analysis could be beneficial to meet the expressed demand for further instruction. The design and implementation of courses dealing with basic computer literacy on one hand and advanced geospatial topics in the context of economics on the other hand form the perspective for further development of the presented approach.

Based on the competencies to be imparted after this proposition, students gain the ability to job-related sustainable economic decisions based on geospatial data and geospatial analytics. They will comprehend relevant aspects of spatial economy for their professional careers. Through this, they can provide literacy about applying geospatial methods and the trustworthiness of geospatially based decisions.

Overall, the unit about geospatial data literacy was successful by imparting core skills of geodata literacy to non-geo-related students. Of course, to work with geodata self-reliantly, further in-depth education would be necessary. But being spatially aware, knowing the basics of spatial reasoning and thinking and the potential of geodata analyses are key competencies for future decision-makers. It enables them to consider and—if appropriate—conduct geodata analyses to reach geospatially based, sound sustainable decisions.

## Data Availability

The statistical results of this study are published here. There are no further data available.
